# Successful Disinfection

**Published:** 1889-05-04

**Authors:** 


### Successful Disinfection.

Messrs. Jeyes, whose disinfectants we have noticed before, have had a flattering success on the other side of the Channel. The Minister of Agriculture appointed a committee to enquire into the best means of disinfecting the cattle market of La Villette, Paris, and neutralising the morbid germs of different diseases which animals might contract. At first, carbolic acid was tried, but the results were not satisfactory. It did not dilute easily, and it left the ground so greasy that complaints were made as to possible danger to the animals by slipping or falling as they moved about. Moreover, it made dark stains upon the pavement, and as it was difficult to make a perfect mixture of the carbolic acid, the effects of it were very irregular. One spot was disinfected beyond its needs while another was left untouched. The odour, too, was so strong that it could not be used in the cowhouses, etc., where it was most needed. After two months' trial, therefore, carbolic acid was discarded, and cresyl (Jeyes' fluid) used in its stead. The report of the committee says that it has acted perfectly. While carbolic acid only masked the ordinary odours by another, cresyl does away with all smells, and results in thorough and satisfactory disinfection.

